# The morphology, genetic structure, and haplotype distribution of the invasive freshwater snails *Biomphalaria straminea* and *Physa acuta* in Guangdong, China

**DOI:** 10.1186/s41182-025-00780-y

**Published:** 2025-07-28

**Authors:** Ping He, Yunyi Hu, Jehangir Khan, Yan Huang, Zhanhong Yuan, Benjamin Sanogo, Du Gao, Jun Liu, De Wu, Jingdiao Chen, Zhongdao Wu, Song Liang, Xi Sun, Datao Lin

**Affiliations:** 1 Medical Department of Xizang, Minzu University, Xianyang, China; 2 Department of Parasitology, Key Laboratory of Tropical Disease Control (Ministry of Education), Zhongshan School of Medicine, Sun Yat-Senen University, Guangzhou, China; 3 Chinese Atomic Energy Agency Center of Excellence on Nuclear Technology Applications for Insect Control, Provincial Engineering Technology Research Center for Diseases-Vectors Control, Sun Yat-Senen University, Guangzhou, China; 4 Department of Zoology, Abdul Wali Khan University Mardan, Mardan, Pakistan; 5 Hainan General Hospital, Hainan Medical University, Haikou, China; 6 Laboratory of Parasitology, Institut National de Recherche en Sante Publique, Bamako, Mali; 7 Clinical Laboratory, Dalian Dermatosis Hospital, Dalian, China; 8 Guangdong Provincial Center for Disease Control and Prevention, WHO Collaborating Centre for Surveillance, Research and Training of Emerging Infectious Diseases, Guangzhou, China; 9 Department of Environmental Health Sciences, School of Public Health & Health Sciences, University of Massachusetts, Amherst, USA

**Keywords:** *Schistosoma mansoni*, *Angiostrongylus cantonensis*, Invasive species, *Biomphalaria straminea*, *Physa acuta*, Mitochondrial haplotype, Disease vector

## Abstract

**Background:**

*Biomphalaria* and *Physa* (order Gastropoda) serve as vectors and reservoirs for infectious agents that affect both humans and animals. This study provides updated insights into the epidemiology, morphology, phylogeny, and haplotype diversity of *Biomphalaria* and *Physa* snails in Guangdong Province, southern China.

**Methods:**

Field surveys were conducted across Guangdong Province from 2016 to 2023. Morphological observations included assessment of snail shape, shell height, and aperture width. Molecular analysis targeted several genes, including Cytochrome c oxidase subunit 1 (*COI*), internal transcribed spacer (ITS), 18S rRNA, 16S rRNA, and 28S rRNA. Evolutionary trees were constructed with neighbor-joining and maximum likelihood methods. Haplotype networks were generated from *COI* sequences collected from multiple geographic locations.

**Results:**

*Physa acuta* was detected in 92% of surveyed sites, showing broad distribution and notable mitochondrial diversity (15 haplotypes). The dominant haplotype (Hap_3) was shared with sequences from Chile and the Netherlands. In contrast, *Biomphalaria straminea* was found at 62% of sites and displayed limited genetic variation (2 haplotypes), despite visible morphological dimorphism (red/black forms). Phylogenetic analysis exhibited minimal differences in 16S rRNA and *COI* gene sequences among turret snail strains, with *B. straminea* clustering closely to South American lineages. Morphometric analyses revealed significant size differences among strains, for example, Shuanglong *B. straminea* had a shell width of 8.74 ± 0.26 mm, whereas Zengcheng *P. acuta* exhibited 11.07 ± 0.90 mm. In contrast, analysis of 28S and 18S rRNA confirmed species boundaries but lacked at the intraspecific level.

**Conclusions:**

Our analysis of multiple target genes confirms that mitochondrial markers (*COI* and 16S rRNA) are effective for studying the evolutionary dynamics of freshwater invasive snails. *Physa acuta* exhibits a widespread distribution and notable genetic diversity across Guangdong, while *B. straminea* shows limited genetic variation, suggesting strong genetic conservation within the species.

**Supplementary Information:**

The online version contains supplementary material available at 10.1186/s41182-025-00780-y.

## Introduction

Freshwater snails of the genera *Biomphalaria* and *Physa* are key intermediate hosts for parasites such as *Schistosoma mansoni* Sambon, 1907 and *Angiostrongylus cantonensis* Chen, 1935, posing significant public health threats in endemic regions [1, 2]. *Biomphalaria straminea*, first detected in Hong Kong in 1974 and later in Guangdong Province, China, has established invasive populations with demonstrated susceptibility to *S. mansoni* [2–6]. Its rapid colonization of South America and Africa underscores its potential to drive schistosomiasis transmission in Guangdong’s urbanizing landscapes [2, 7–9]. Concurrently, *Physa acuta*, a globally invasive bladder snail associated with angiostrongyliasis and echinostomiasis, was first reported in China in the early 2000s and has since spread widely [10–14]. Despite their medical relevance, critical gaps remain in understanding the genetic diversity and haplotype structure of these snails in Guangdong, where anthropogenic change and climate driven habitat expansion may accelerate their spread [3, 13].

Current knowledge of *B. straminea* in China is largely based on fragmented morphological surveys, while molecular insights into its genetic conservation or divergence remain limited [8, 15]. Similarly, for *P. acuta*, despite its widespread detection, phylogenetic relationships among Chinese and global populations are poorly resolved, obscuring its invasion history and potential for secondary introductions [10, 16].

Previous studies have emphasized the need for an integrative approach employing multi-locus genetic markers (e.g., *COI*, ITS, rRNA) and haplotype network analysis to elucidate origins, dispersal routes, and adaptive traits [2, 9, 11]. Such methods are critical for identifying genetic bottlenecks, hybrid zones, or cryptic diversity that may influence vector competence and disease transmission dynamics [4, 17].

This study aims to fill these gaps by systematically analyzing the distribution, morphology, and genetic structure of *B. straminea* and *P. acuta* across Guangdong Province, China. By combining mitochondrial and nuclear markers, we reconstruct phylogenetic relationships and assess haplotype diversity. By incorporating global GenBank sequences, we contextualize Guangdong’s populations within broader biogeographic patterns. Our results aim to inform targeted surveillance strategies for invasive snails and may contribute to mitigating the emergence of snail-borne diseases in China.

## Methods

### Field sampling and morphological characterization

Systematic field surveys were conducted from 2016 to 2023 across 21 pre-selected sites in Guangdong Province, China, covering diverse aquatic habitats including rivers, streams, floodways, and shallow pools. Sampling sites were stratified based on historical records of *B. straminea* and *P. acuta* distributions [2], or, alternatively, sites were randomly selected for the survey, with GPS coordinates recorded for each location (Tables 1, 2). Specimens were collected by two trained researchers using standardized dip-netting protocols, transported live to the laboratory, and stored at −80 °C for subsequent analyses. Morphometric parameters (shell height, width, aperture dimensions) were measured using digital Vernier calipers (precision ± 0.01 mm) following established methods [2, 18]. Phenotypic variations, including color morphs (e.g., red versus black *B. straminea*), were documented to assess intraspecific diversity.

**Table 1:** Geographic distribution and morphological characteristics of *Biomphalaria straminea* populations sampled in Guangdong Province, China (2016–2023)

Locality	Geographic coordinates (E, N)		Specimen code	Phenotype	Habitat type
Dongguan	E114°07′72″	N22°91′60″	Dongguan_17011-3	Black	Stream
	E114°07′72″	N22°91′60″	Dongguan_red1,10,44	Red	Shallow pool
	E114°09′58″	N22°99′41″	Dongguan_17021-3	Black	River
	E114°09′58″	N22°99′41″	Dongguan_17021-3	Red	River
	E114°10′76″	N23°03′73″	Dongguan_17031-3	Black	River
	E114°05′48″	N22°79′66″	Empty shell	–	River
Shenzhen	E114°14′90″	N22°55′46″	Luohu_1-3	Black	Floodway
	E113°94′94″	N22°57′77″	Dashahe_12,13,33	Black	River
	E114°04′22″	N22°71′65″	Baoan_38, Baoan_40	Black	Stream
	E114°04′22″	N22°71′65″	Baoan_red2, 3	Red	Stream
	E114°06′43″	N22°36′01″	Baoan_Yuebao_B4	Black	Stream
	E114°16′23″	N22°43′48″	Shuanglong_1-3	Black	River
	E114°16′23″	N22°43′48″	Shuanglong_red1	Red	River
	E114°24′14″	N22°36′55″	Tuyang_1-3	Black	River
	E114°24′35″	N22°38′06″	Kuiyong_1-3	Black	River
	E114°34′79″	N22°69′19″	Pingshan_1-3	Black	River
	E114°28′21″	N22°35′49″	Dapeng_1-3	Black	River
	E114°15′26″	N22°44′05″	No found	–	River
Guangzhou	E113°48′55″	N23°17′49″	No found	–	River
	E113°17′59″	N23°5′54″	No found	–	Ditch
Huizhou	E114°48′26″	N22°89′78″	Huiyang_1-3	Black	River
	E114°41′83″	N22°81′58″	No found	–	River
Qingyuan	E112°58′09″	N24°30′85″	No found	–	River
Zhaoqing	E111°49′34″	N23°41′15″	No found	–	Shallow pool
Yunfu	E111°44′55″	N23°52′49″	No found	–	River

**Table 2:** Sampling sites and habitat preferences of *Physa acuta* coexisting with *Biomphalaria straminea* in Guangdong Province

Locality	Geographic coordinates (E, N)		Specimen code	Habitat type	Co-occurrence with *B. straminea*
Guangzhou	E113°48′55″	N23°17′49″	Zengcheng_1-4	River	No
	E113°17′59″	N23°5′54″	Haizhu_1-5	Ditch	No
Shenzhen	E114°16′23″	N22°43′48″	Shuanglong_1-3	River	Yes
	E114°15′26″	N22°44′05″	Longyuan_1-3	River	Yes
	E114°24′35″	N22°38′06″	Kuiyong_1-3	River	Yes
	E114°34′79″	N22°69′19″	Pingshan_1-3	River	Yes
	E114°28′21″	N22°35′49″	Dapeng_1-3	River	Yes
	E114°24′14″	N22°36′55″	No found	River	No
	E114°14′90″	N22°55′46″	Luohu_1-3	Floodway	Yes
Dongguan	E114°09′58″	N22°99′41″	17021-3	River	Yes
	E114°05′48″	N22°79′66″	No found	River	No
	E114°07′72″	N22°91′60″	17011-3	Stream	Yes
Yunfu	E111°44′55″	N23°52′49″	Yunfu_1-3	River	No
Qingyuan	E112°58′09″	N24°30′85″	Qingyuan_1-3	River	No
Zhaoqing	E111°49′34″	N23°41′15″	Zhaoqing_1-3	Shallow pool	No

*P. acuta* exhibited broader habitat tolerance than *B. straminea*, colonizing ditches and floodways

### DNA extraction and PCR amplification

Total genomic DNA was extracted from the head–foot tissue of 48 *P. acuta* and 44 *B. straminea* specimens using the HiPure DNA Mini Kit (Magen, China), with purity (A260/A280 > 1.8) and concentration verified via Nanodrop spectrophotometry (Thermo Scientific, USA). Six molecular markers were amplified: mitochondrial cytochrome c oxidase subunit I (*COI*) and 16S rRNA for both species, supplemented by nuclear ITS, 18S rRNA, and 28S rRNA for *P. acuta*.

The PCR primers used for *Biomphalaria* snails targeted the *COI* gene, 16S rRNA gene and the entire ITS [19] (which includes the 5.8S rDNA gene together with the flanking ITS1 and ITS2 spacers) (Table S1). For *P. acuta*, the primers targeted the *COI* gene, ITS gene, 16S rRNA gene, 18S rRNA gene, and 28S rRNA gene (Table S2). The PCR amplification conditions (using Takara Ex Taq Master Mix) for snails are provided in Tables S3, S4, S5. Products were visualized on 1.5% agarose gels, purified (TaKaRa gel extraction kit), and sequenced bidirectionally by Majorbio (Guangzhou, China).

### Phylogenetic and haplotype network analyses

Sequences were aligned using the ClustalW algorithm implemented in MEGA7 (v7.0.18) [20], with manual adjustment for indels. The best-fit nucleotide substitution model (GTR + G) was selected using jModelTest2 based on the Akaike Information Criterion (AIC) [17]. Phylogenetic trees were reconstructed using both neighbor-joining (NJ, 1,000 bootstrap replicates) and maximum likelihood (ML) methods in RAxML v8.2.12 under the GTR + G model to validate topological robustness [17]. For haplotype inference, *COI* sequences from this study were combined with globally representative GenBank entries (e.g., *P. acuta*: Chile, Cuba, USA; *B. straminea*: Brazil, Venezuela) filtered for ≥ 98% coverage and ≤ 5% missing data. Haplotype networks were generated in PopART (v1.7) using the Templeton–Crandall–Sing (TCS) algorithm to visualize genetic connectivity [16, 21].

### Statistical analysis

Pairwise genetic distances were calculated in MEGA7, and morphological variations were analyzed via one-way ANOVA (SPSS v19.0), with significance thresholds set at *P* < 0.05.

## Results

### Distribution and habitat overlap of invasive snails

**Fig. 1:**
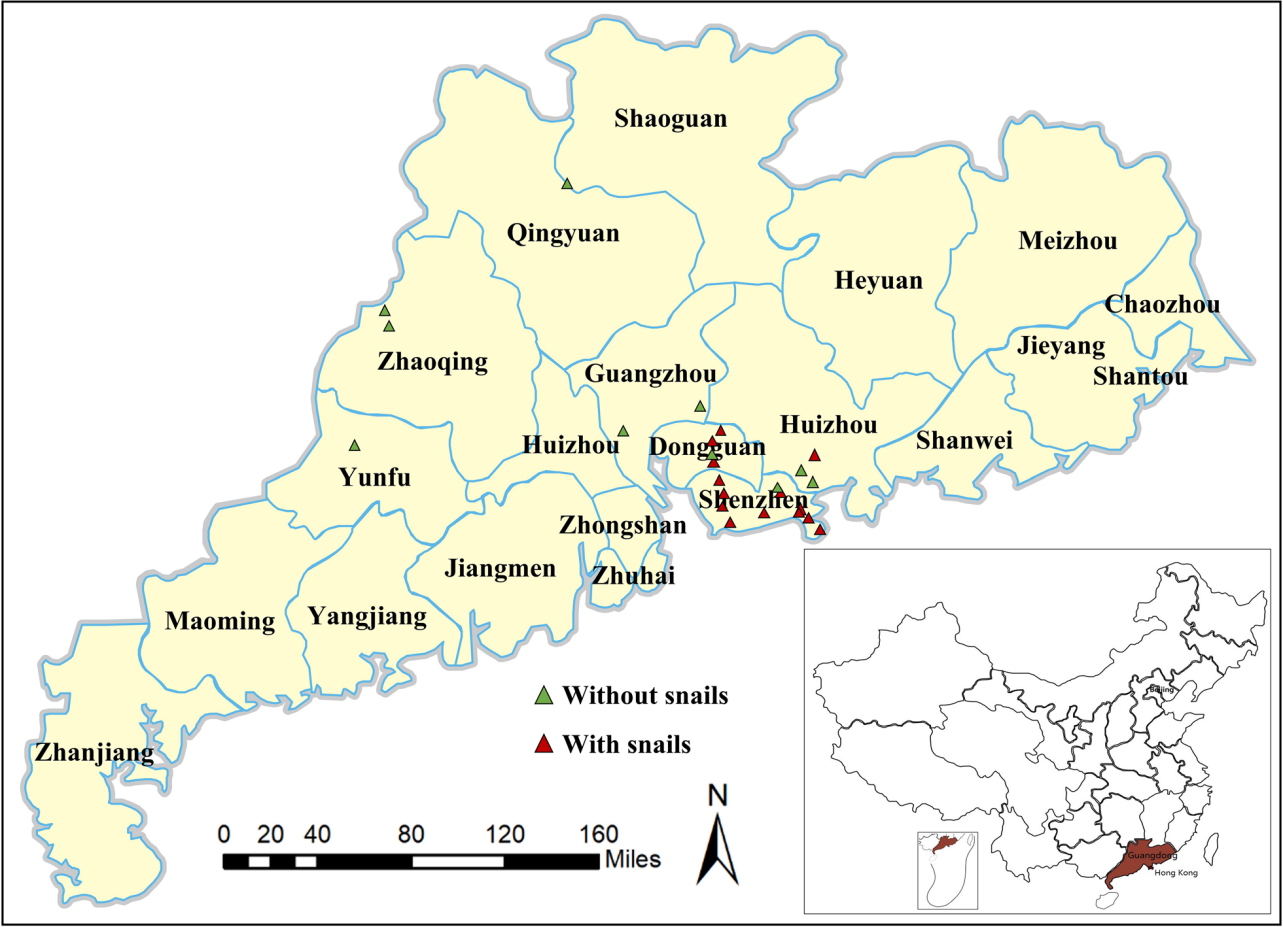
Distribution of snails in Guangdong, China. All surveyed sites are marked on the map. Green triangles: sites where no snails were found; red triangles: sites where snails were found

**Fig. 2:**
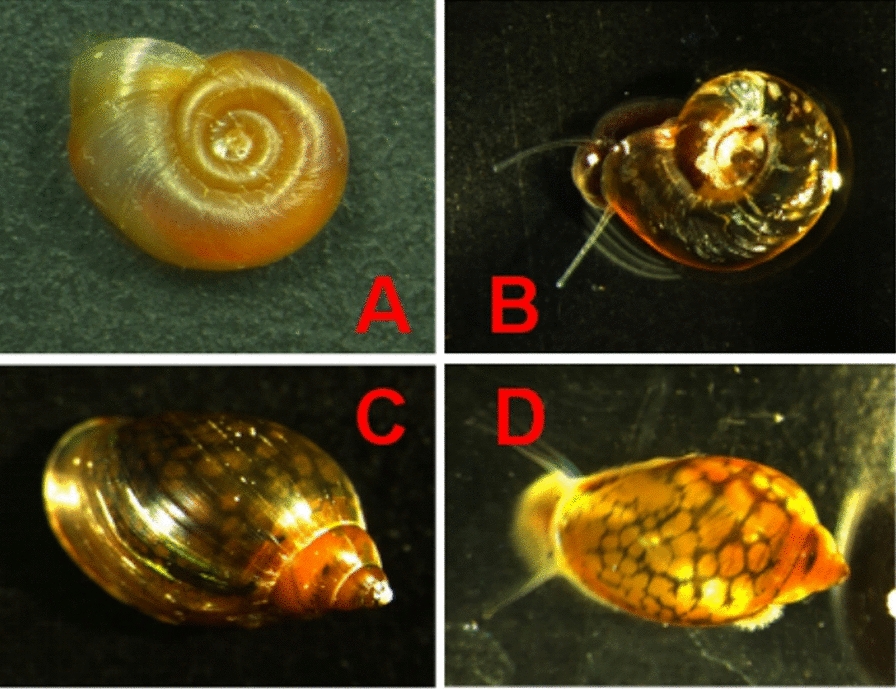
Phenotypic variation in Biomphalaria straminea (**A**, **B**) and Physa acuta (**C**, **D**)

Field surveys across Guangdong Province identified *B. straminea* at 61.9% (13/21) of sampled sites, with hotspots in Shenzhen, Dongguan, and Huizhou (Table 1, Fig. 1). Notably, two distinct color morphs, red and black, were observed in *B. straminea* populations from Shenzhen and Dongguan, coexisting in slow-flowing rivers rich in aquatic vegetation (Fig. 2A, B). *P acuta* displayed broader ecological plasticity, colonizing a variety of aquatic habitats such as rivers, ditches, and floodways across six cities, including areas where *B. straminea* was absent (Tables 1, 2). Co-occurrence of both species was recorded in 38% of sites, primarily in nutrient-rich lowland rivers. Morphological features of *P. acuta* included a slender, elongated body and a smooth, glossy shell, with some shells having a darker color and others lighter (Fig. 2C, D).

### Morphological divergence and phenotypic plasticity

**Fig. 3:**
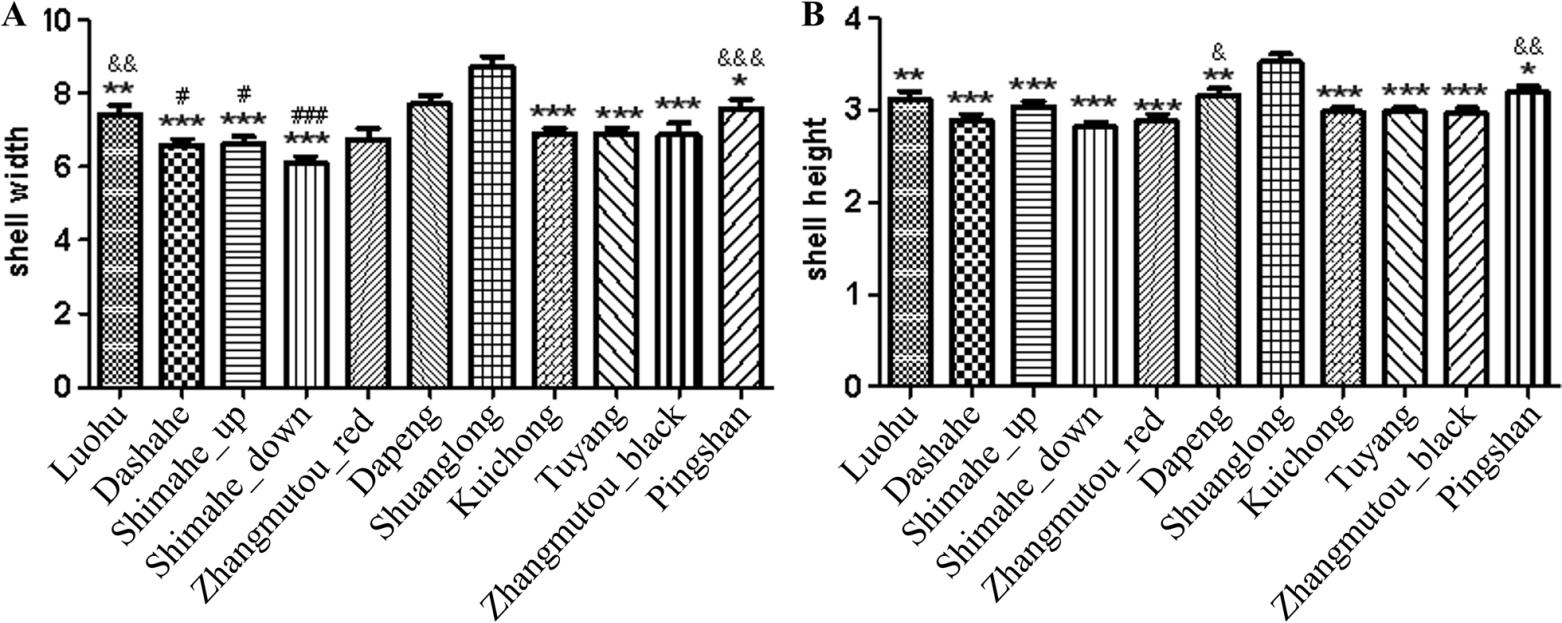
Shell size variation among geographically distinct Biomphalaria straminea populations. ## represents a significance compared with the Dapeng group (*P* < 0.01). ** represents a significance compared with the Shuanglong group (*P* < 0.01). & represents a significance compared with the Shima River downstream group (*P* < 0.05)

**Fig. 4:**
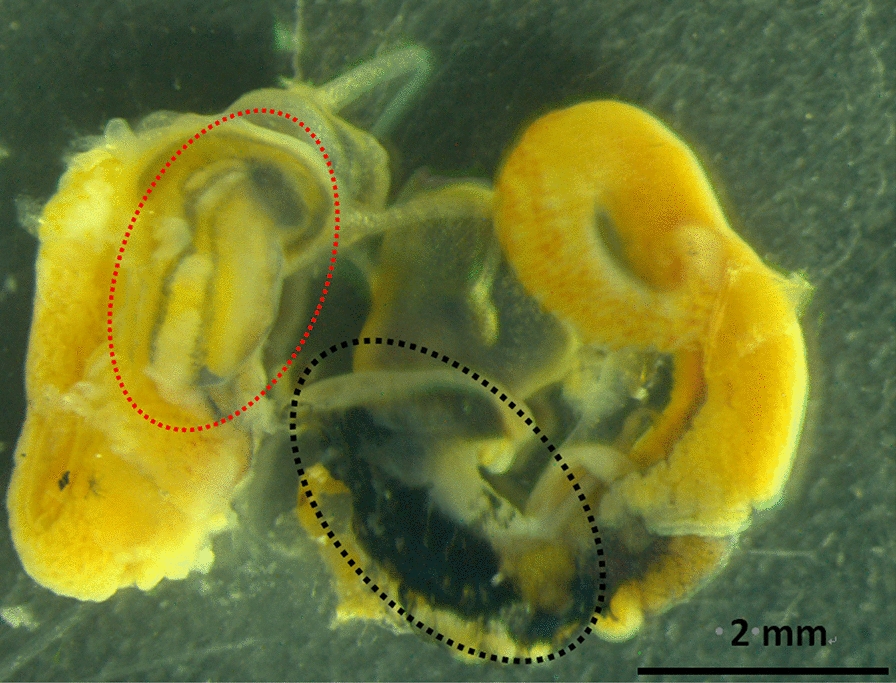
Soft tissue pigmentation in color variants of B. straminea. Red Biomphalaria snail (left) and blank Biomphalaria snail (right). Prostatic branch counts ranged from 10 to 19 across sampling points, consistent with the morphological classification criteria for B. straminea (10–20 prostatic branches) [9]

Significant size variations were observed among *B. straminea* populations, with shell width diameters ranging from 4.34 to 10.84 mm and height from 2.00 to 4.10 mm) (Fig. 3). The Shuanglong strain (Shenzhen) had the largest shell width (8.73 ± 0.26 mm), differing significantly from Pingshan (Δ = 1.16 mm, *P* < 0.05) and Shima River groups (Δ = 2.00 mm, *P* < 0.05). Red morphs exhibited visibly reduced melanin deposition compared to black morphs (Fig. 4), supporting a genetic basis for pigmentation variations [22].

**Fig. 5:**
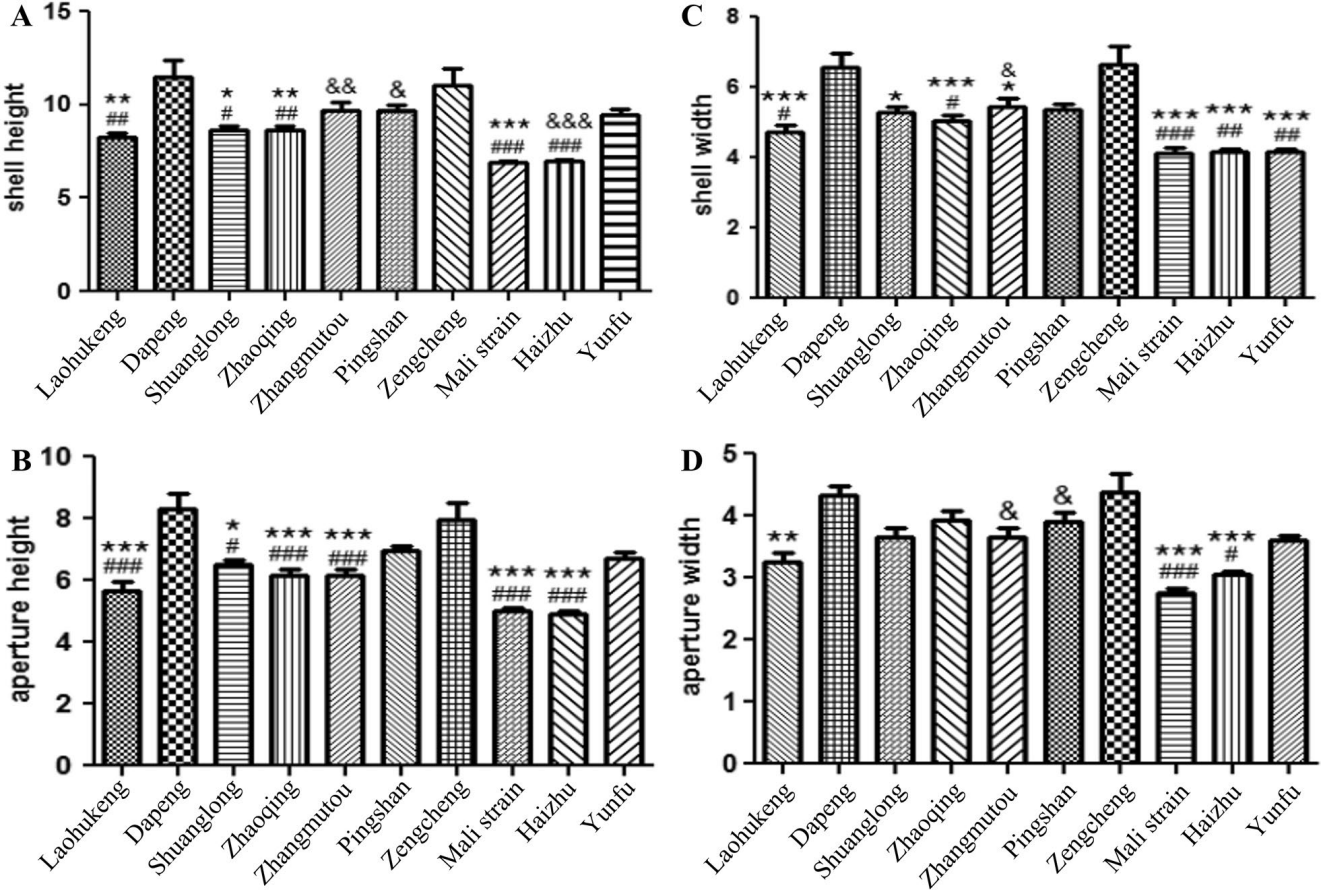
Geographical variation in Physa acuta shell dimensions. ## represents a significance compared with the Dapeng group (*P* < 0.01). ** represents a significance compared with the Zengcheng group (*P* < 0.01). & represents a significance compared with Mali group (*P* < 0.05)

For *P. acuta*, shell height ranged from 6.50 to 14.20 mm and width from 3.46 to 8.80 mm (Fig. 5). The Zengcheng (Guangzhou) and Dapeng (Shenzhen) populations showed the largest shell dimensions, although differences were not statistically significant (Δ = 0.43 mm,* P* > 0.05), potentially reflecting local adaptation to environmental factors.

### Phylogenetic conservation in *Biomphalaria straminea versus* genetic diversity in* Physa acuta*

**Fig. 6:**
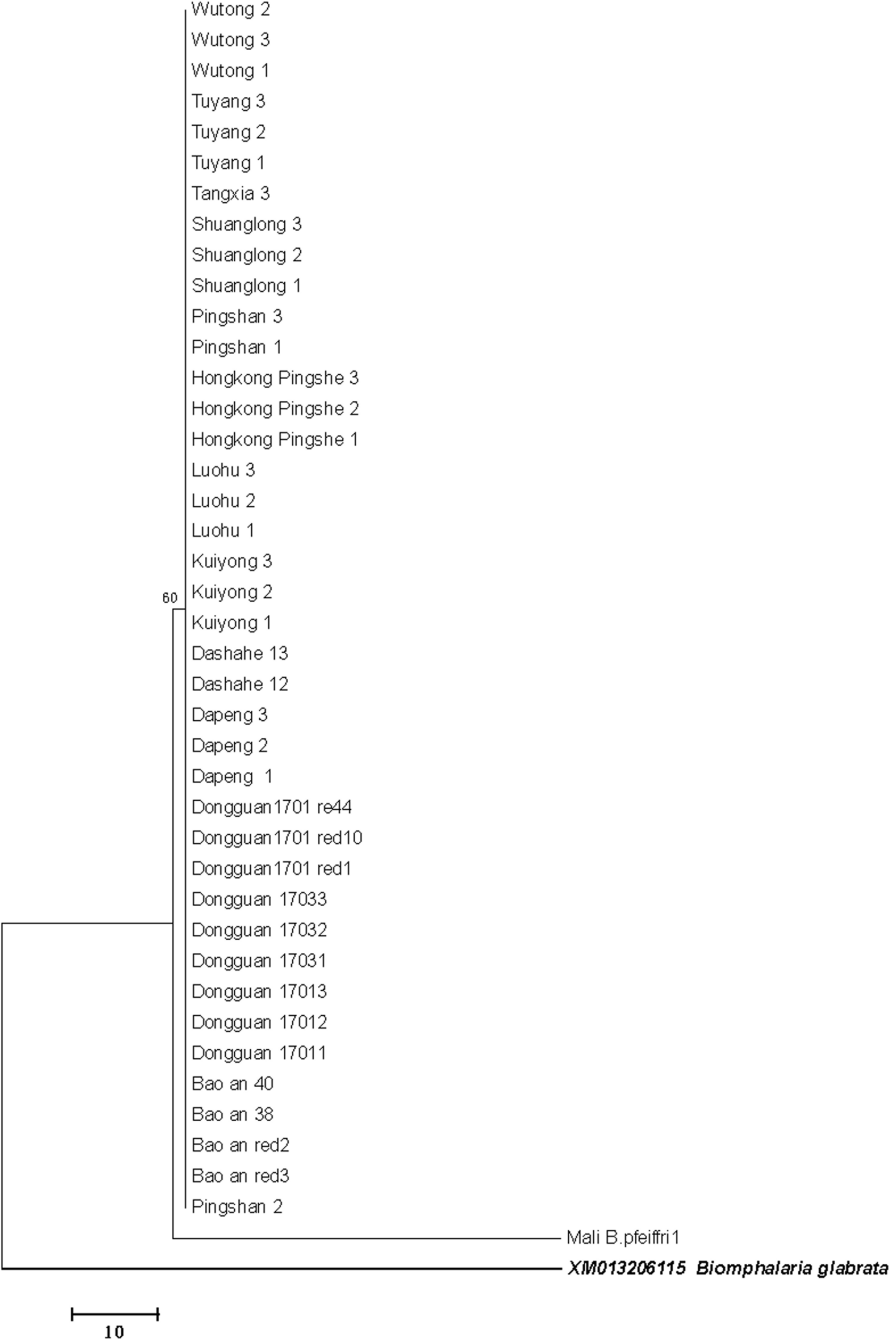
Neighbor-joining phylogeny of Biomphalaria straminea based on mitochondrial COI sequences

**Fig. 7:**
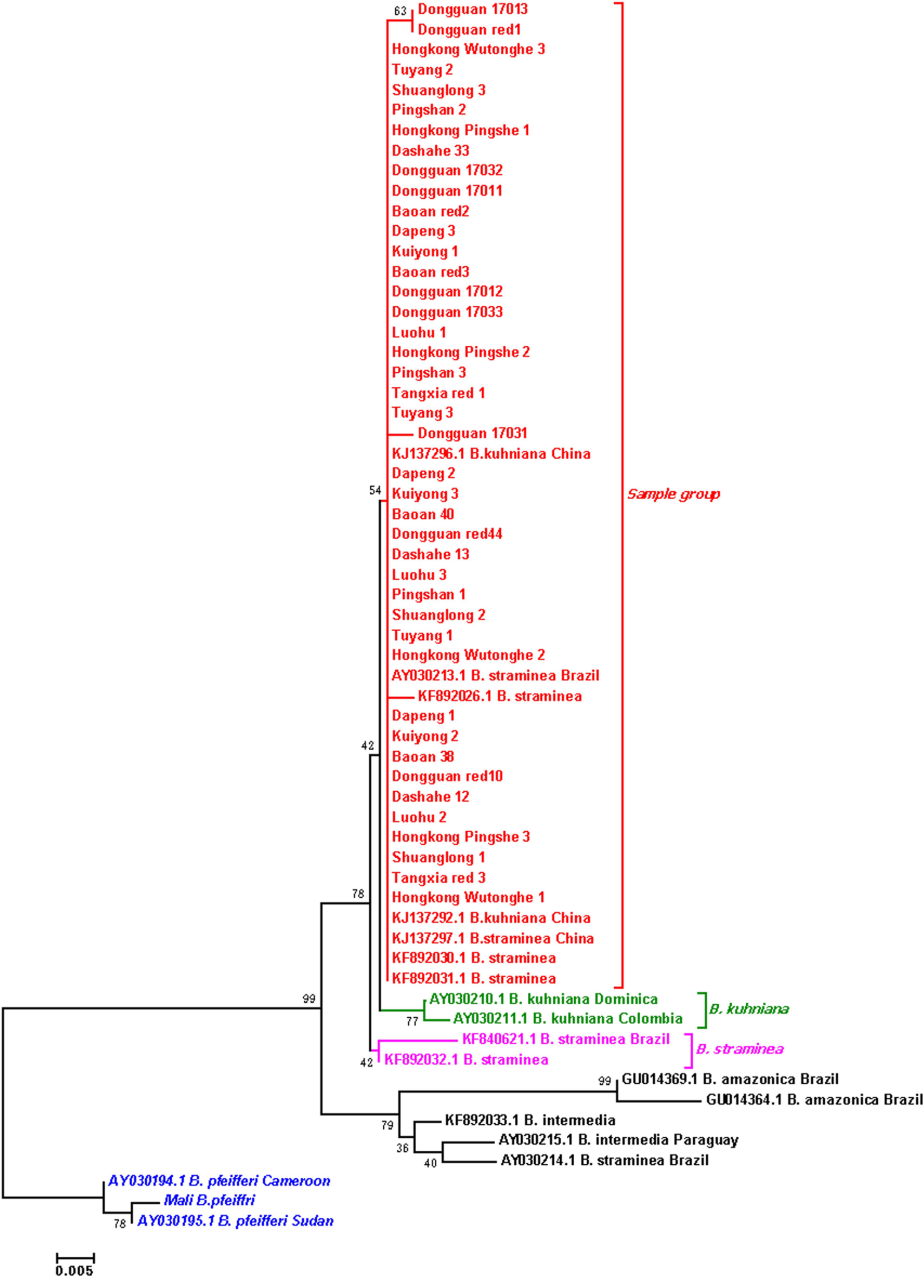
16S rRNA phylogeny of Biomphalaria straminea in Guangdong, constructed using the neighbor-joining method

Mitochondrial *COI* and 16S rRNA analyses revealed minimal genetic divergence among *B. straminea* populations (intragroup distance = 0.000 for *COI*, 0.004 for 16S rRNA), clustering closely with South American conspecifics (Brazil, Venezuela) but distinct from *B. pfeifferi* (*COI* distance = 1.433; Figs. 6, 7, Fig. S1). ITS-based phylogenies constructed using both neighbor-joining and maximum likelihood methods further supported this pattern (Figs. S2-3).

**Fig. 8:**
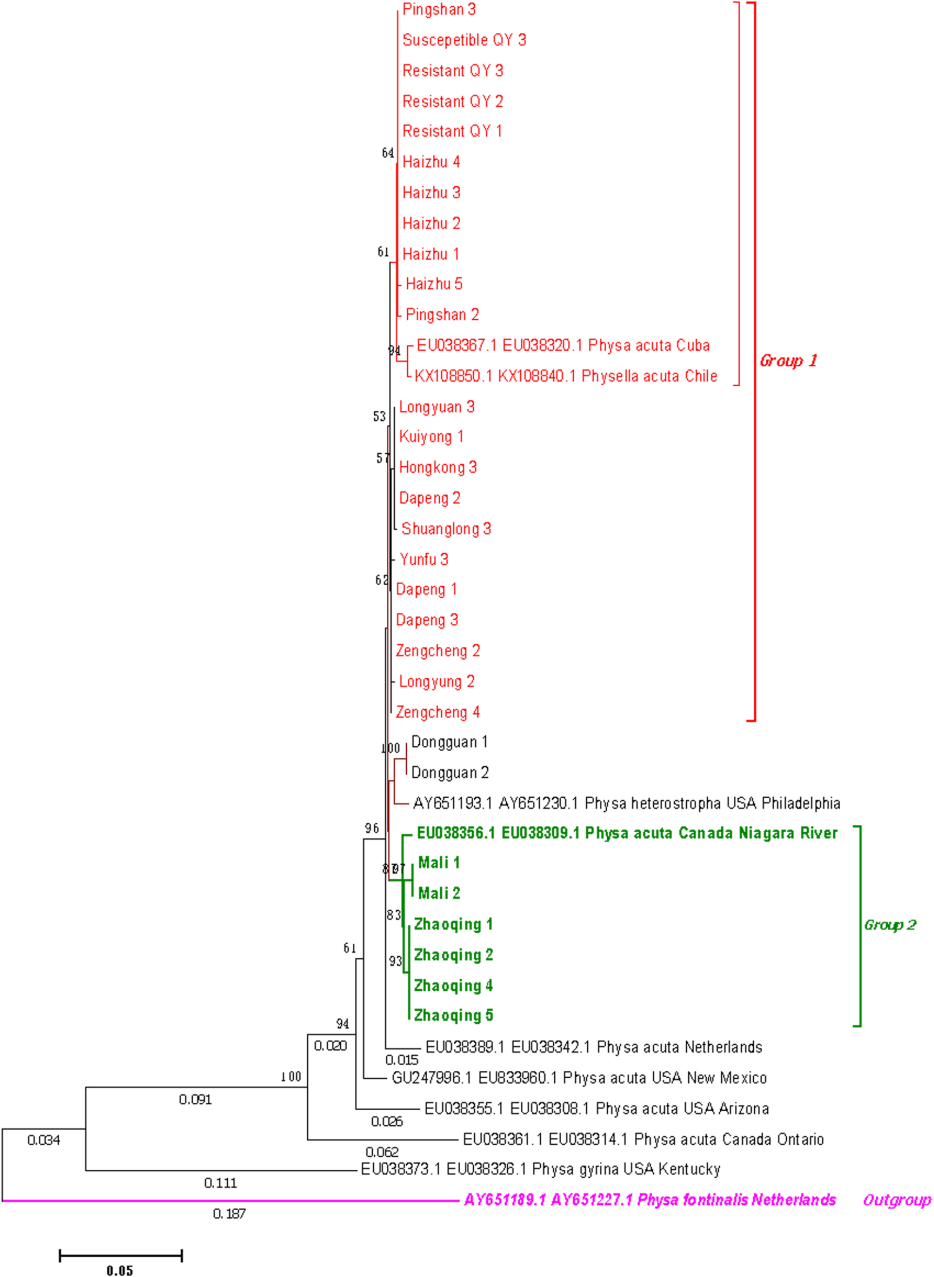
Concatenated COI-16S rRNA phylogeny of Physa acuta in Guangdong, generated by the neighbor-joining method

**Fig. 9:**
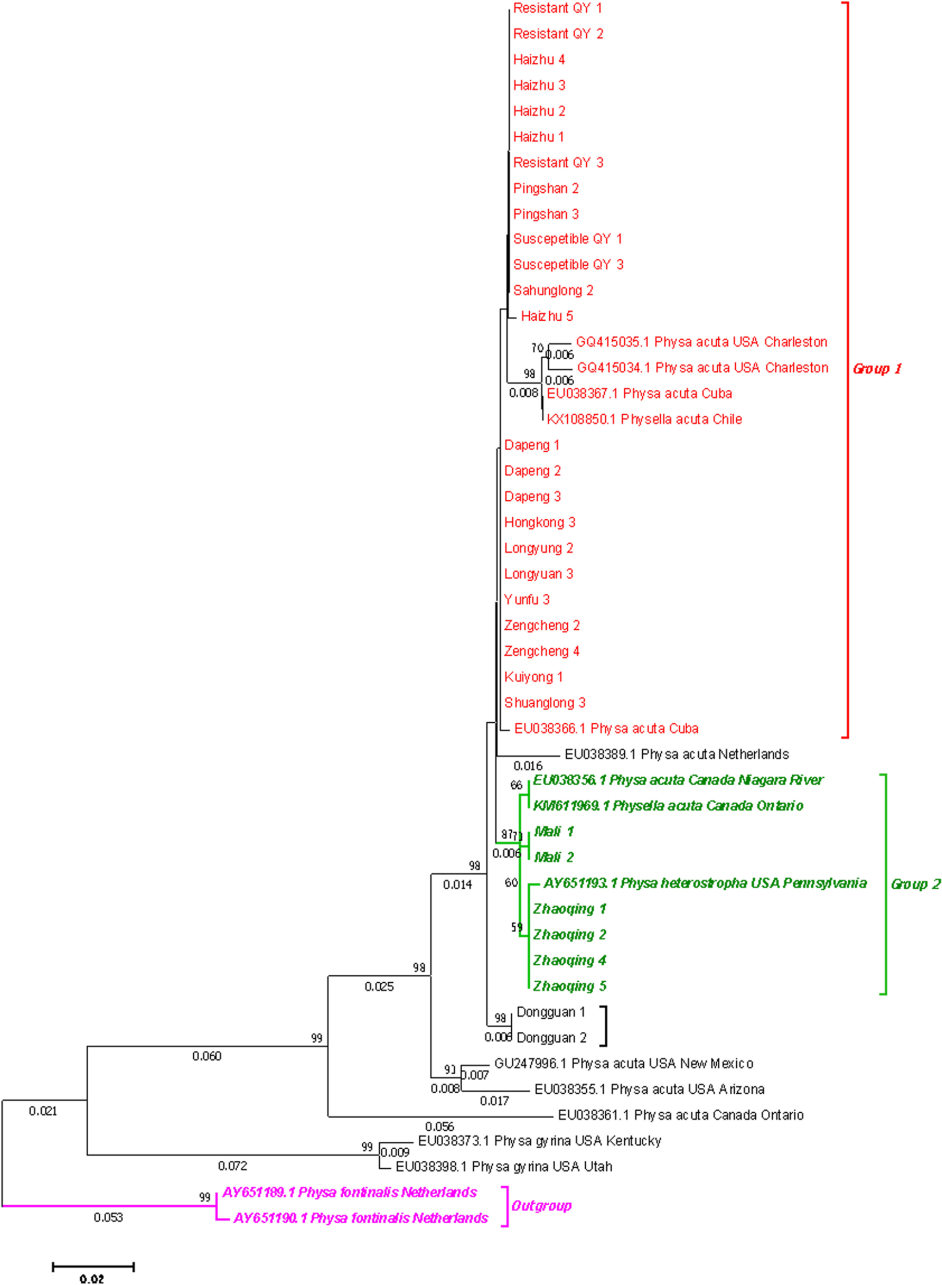
COI phylogeny of Physa acuta in Guangdong, constructed using the neighbor-joining method

**Fig. 10:**
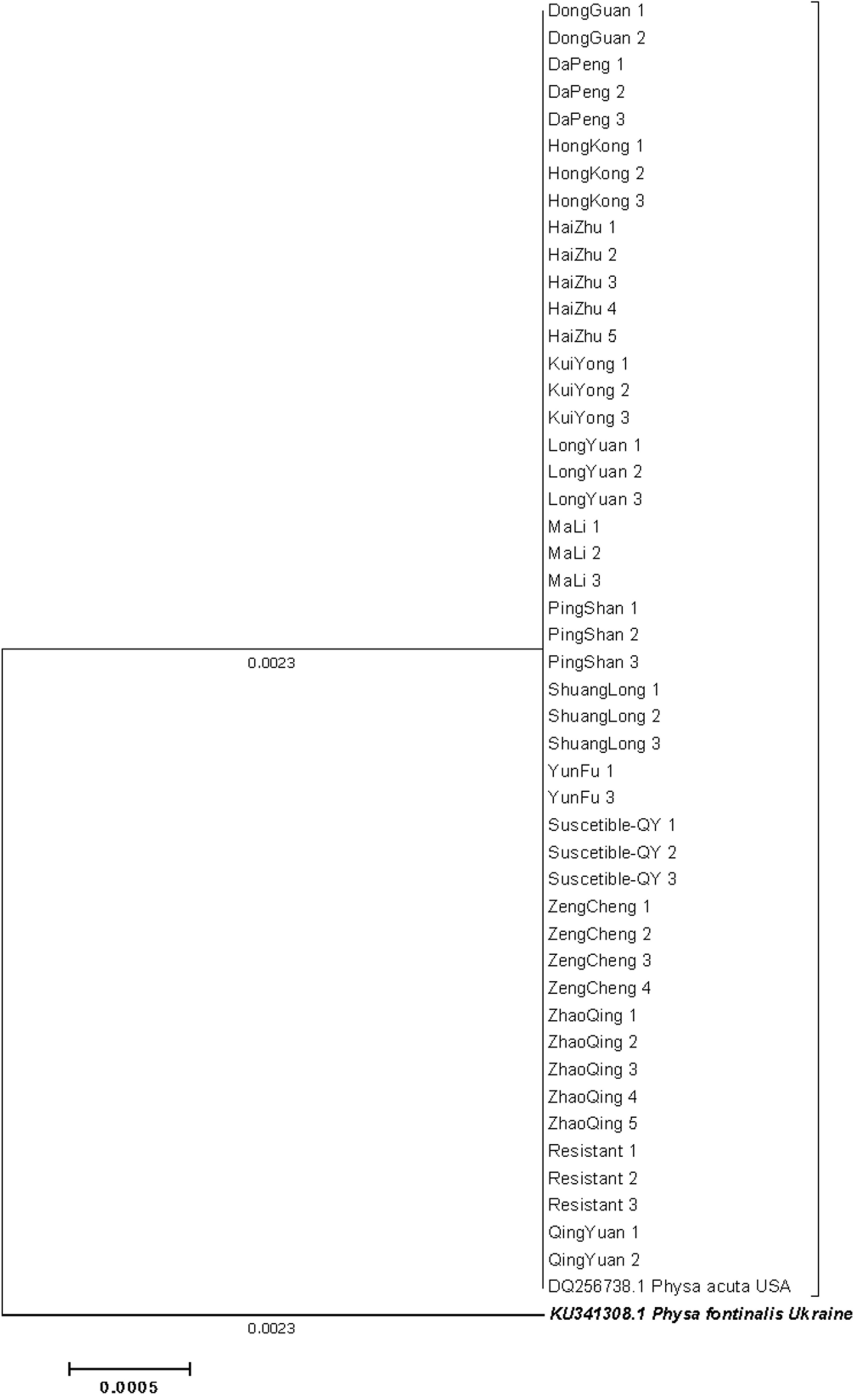
28S rRNA phylogeny of Physa acuta in Guangdong busing neighbor-joining method

**Fig. 11:**
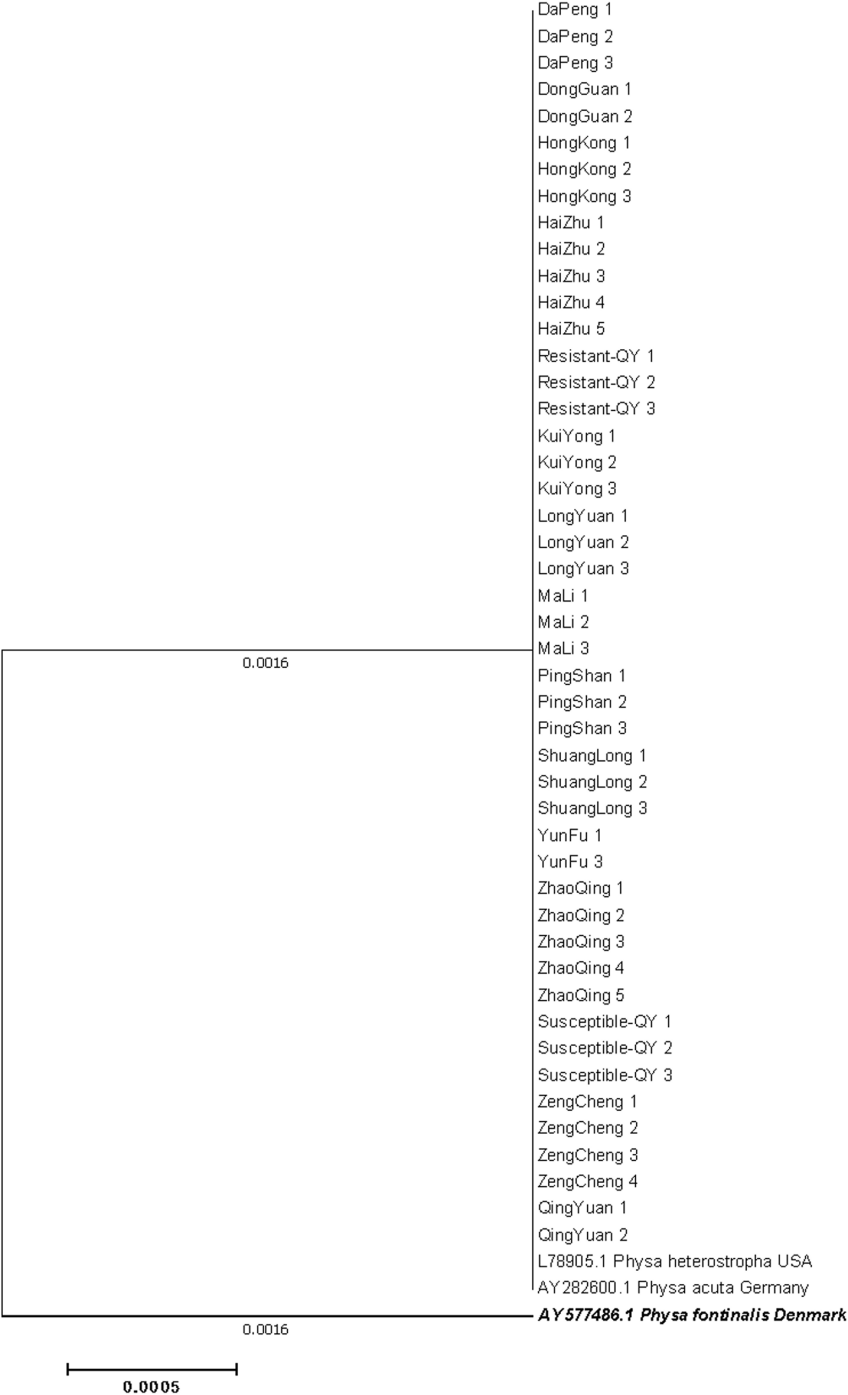
18S rRNA phylogeny of Physa acuta in Guangdong, generated by neighbor-joining method

**Fig. 12:**
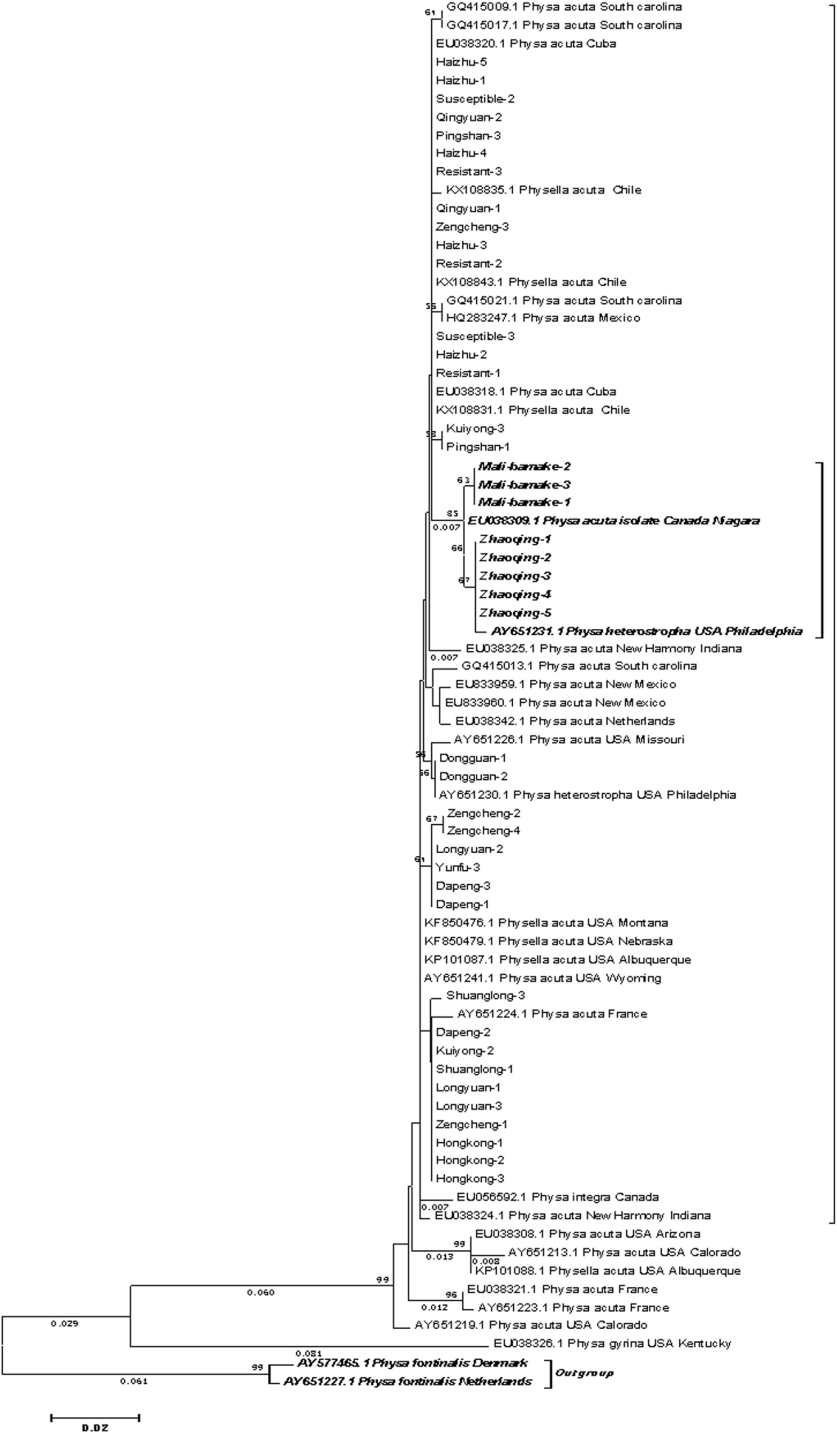
16S rRNA phylogeny of Physa acuta in Guangdong, generated by neighbor-joining method

**Fig. 13:**
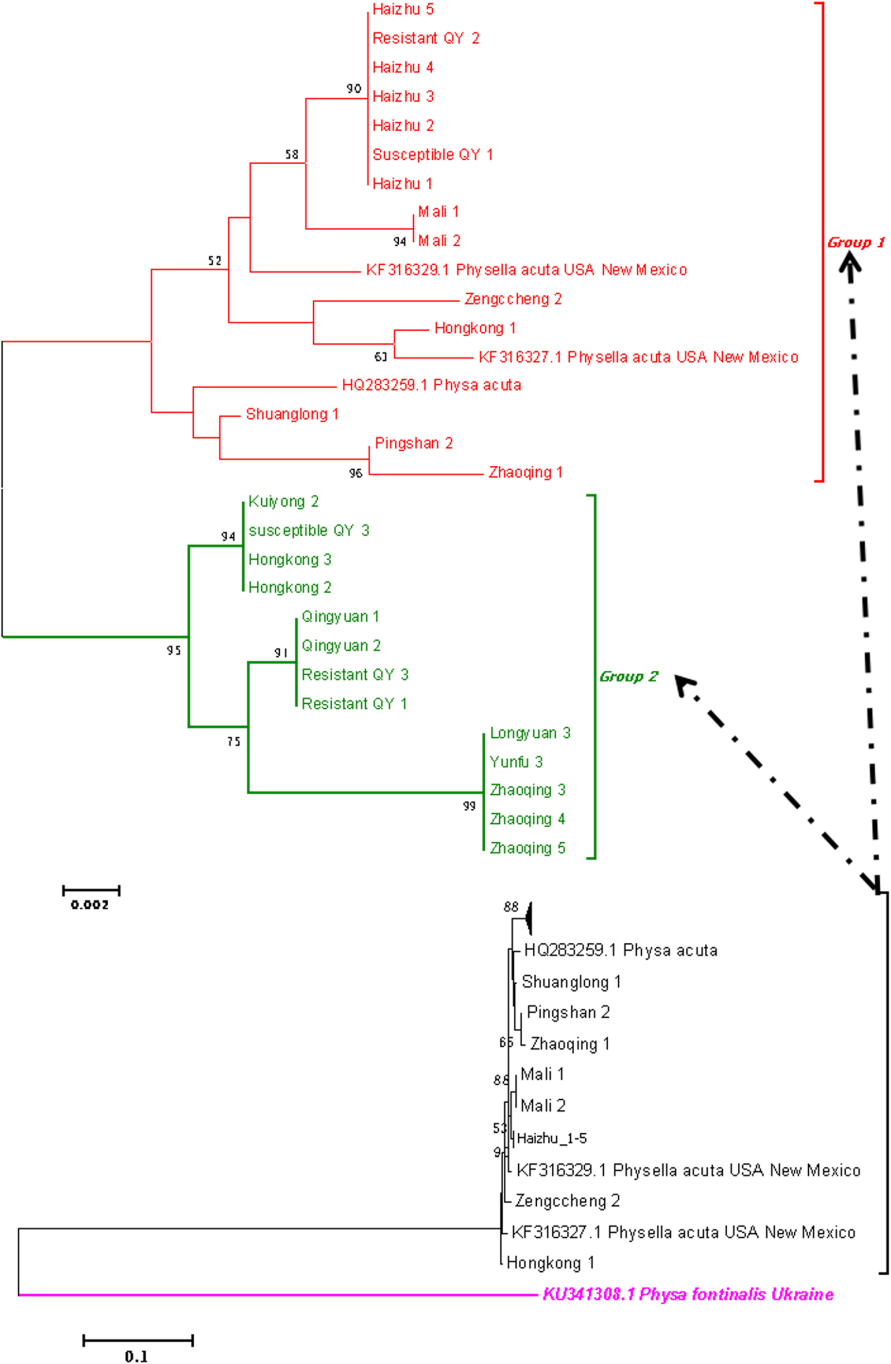
ITS-based phylogeny of Physa acuta in Guangdong, constructed using the neighbor-joining method

In contrast, *P. acuta* showed considerable geographic genetic diversity. Concatenated *COI*-16S rRNA phylogenies identified two major clades (Fig. 8): 63% of Guangdong strains clustered with Cuban and Chilean lineages (genetic distance < 0.007), while 37% grouped with North American haplotypes (Fig. 9). Nuclear markers (ITS, 28S rRNA) confirmed species identity but failed to resolve fine-scale population structure (Figs. 10, 11, 12, 13, Figs. S4-6), highlighting discordance between mitochondrial and nuclear marker resolution.

### Haplotype networks of freshwater invasive snails

**Fig. 14:**
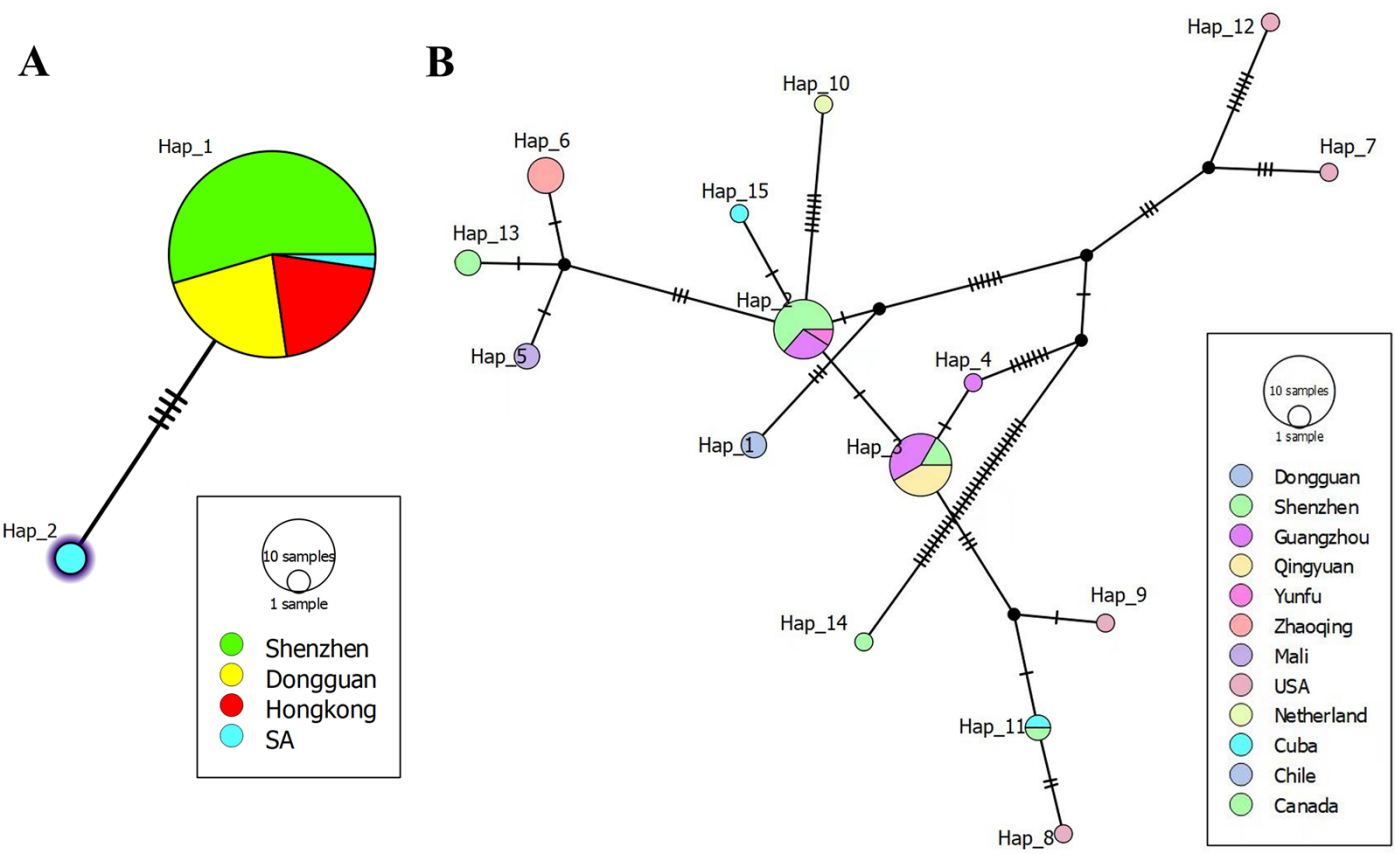
Haplotype networks of COI sequences for **A** B. straminea and **B** P. acuta. **A** Two haplotypes (Hap_1 dominant) with 4 mutational steps separating Guangdong and South American (SA) lineages. B Fifteen haplotypes in P. acuta, with Hap_3 shared globally (China, Chile, Netherlands). The circle area corresponds to haplotype frequency. SA: South America

Haplotype analysis based on the *COI* sequences revealed 15 haplotypes in *P. acuta* (Fig. 14). Hap_3 was the most dominant haplotype, shared by populations from China (Guangzhou, Qingyuan, Yunfu, and Zhaoqing), Chile, and the Netherlands, suggesting widespread global connectivity. Other haplotypes (e.g., Hap_15, Hap_10, Hap_6, and Hap_4) were also shared between international and Chinese populations, while some (Hap_7, Hap_8, Hap_9, and Hap_12) appeared geographically restricted, notably to the USA, indicating possible regional isolation or divergence.

In *B. straminea*, two closely related haplotypes were detected (Fig. 13A). Hap_1 was dominant and present in samples from Shenzhen, Dongguan, and Hong Kong, as well as several South American sites. Hap_2, restricted to South America (SA), differed from Hap_1 by two mutational steps. This limited haplotype diversity supports the hypothesis of a recent, narrow genetic introduction of *B. straminea* into China.

## Discussion

This study provides critical insights into the morphology, phylogeny, and haplotype distribution of two invasive freshwater snails in Guangdong Province, China. Our findings reveal contrasting biological characteristics between *P. acuta* and *B. straminea*, with potential implications for disease surveillance and control strategies.

### Genetic structure and divergence in *Physa acuta*

While *P. acuta* exhibited broad morphological consistency with global populations (shell height: 6.50–14.20 mm; width: 3.46–8.80 mm) [23–25], while mitochondrial *COI*-16S rRNA phylogenies revealed two geographically structured clades. Over 60% of Guangdong strains clustered with Cuban and Chilean lineages, while others grouped with North American *P. acuta*. This pattern is further supported by the haplotype network, where Hap_3, a dominant haplotype, was shared among populations in China, the Netherlands, and Chile [16, 26]. These results challenge earlier assumptions of a single East Asian invasion route [25], suggesting more complex introduction scenarios, possibly involving multiple independent events.

Although we detected 15 *COI* haplotypes among Guangdong *P. acuta* samples, we recognize that haplotype richness alone is insufficient to infer cryptic speciation. Instead, the observed mitochondrial diversity likely reflects population structuring driven by historical introductions. In contrast, nuclear markers (ITS, 18S, 28S rRNA) showed markedly lower variation and failed to distinguish geographic populations. This discrepancy reflects differences in mutation rates and inheritance patterns between mitochondrial and nuclear genomes. Mitochondrial DNA, with its rapid evolution and maternal inheritance, is more sensitive to recent demographic changes. Conversely, nuclear markers, particularly ribosomal genes, evolve slowly and may not detect fine-scale divergence, especially in recently introduced or admixed populations. Thus, while mitochondrial markers revealed regional structuring, the limited variation in nuclear loci supports low species-level divergence and suggests all sampled populations likely belong to the same biological species. To clarify lineage boundaries and potential cryptic diversity, future studies should integrate genome-wide nuclear data and reproductive compatibility assays.

### Genetic homogeneity in *Biomphalaria straminea*: implications for schistosomiasis risk

Despite evident morphological plasticity (red/black color morphs and shell size variations), *B. straminea* populations in Guangdong displayed striking genetic homogeneity, clustering closely with South American conspecifics. The dominance of Hap_1 (98% of samples), shared with Brazilian and Venezuelan lineages, supports the hypothesis of a single successful introduction event, likely via maritime trade in the 1970s [2, 5, 6]. Red morphs, associated with reduced melanin synthesis [22], were observed alongside black phenotypes in high-risk zones (Shenzhen, Dongguan). While prior studies in congeneric species suggest a possible link between albinism and increased susceptibility to *S. mansoni* [2, 22], our findings remain observational and lack supporting infection data. Therefore, any association between color morphs and parasite susceptibility is speculative and warrants further experimental validation.

*COI* and 16S rRNA analyses revealed minimal genetic divergence, confirming a close affinity with South American *B. straminea*. This homogeneity, despite morphological variation, highlights the limitations of relying solely on mitochondrial markers or external morphology for population structure inference [19, 27]. Furthermore, we acknowledge that the exclusion of nuclear markers—such as ITS2 and 28S rRNA—due to amplification failure, limited our ability to achieve high-resolution phylogenetic reconstruction. As a result, concatenated nuclear–mitochondrial datasets, which are essential for detecting cryptic diversity or hybridization, could not be generated. Previous studies have demonstrated the value of multi-locus approaches in resolving evolutionary relationships within the *Biomphalaria* genus [9, 19, 28–31]. Future work should prioritize incorporating nuclear markers to clarify the evolutionary dynamics, population connectivity, and hidden diversity in *B. straminea* across China. Nevertheless, this study demonstrates that mitochondrial sequencing remains a practical and cost-effective approach for the initial surveillance of invasive snail populations.

In our 16S rRNA phylogeny (Fig. 7), *B. kuhniana* clustered closely with *B. straminea* but was genetically distinct from Chinese *B. straminea*. Morphological examination, including prostate branch analysis, confirmed the identification of Chinese specimens as *B. straminea* [2]. Brazilian *B. straminea* sequences included in the haplotype network and phylogenetic analyses clustered with Chinese samples, consistent with previous reports [5, 6]. However, the close genetic proximity between *B. kuhniana* and *B. straminea*, coupled with overlapping morphological traits, raises the possibility of cryptic speciation or unresolved taxonomic boundaries within the complex. Broader genomic datasets are needed for comprehensive taxonomic revision to clarify species delimitation and address possible misidentifications in South American reference samples.

### Public health priorities and future directions

The co-occurrence of both snail species in 38% of sampled sites, particularly nutrient-rich rivers, poses dual risks for *S. mansoni* and *A. cantonensis* transmission. While *B. straminea*’s genetic uniformity may simplify control strategies, *P. acuta*’s mitochondrial diversity demands targeted monitoring of lineages linked to higher invasion success. Further studies should prioritize infection trials to assess vector competence across *P. acuta* haplotypes, high-resolution genomic analysis of pigmentation-related genes in *B. straminea* morphs, and broader surveillance using environmental DNA (eDNA) and remote sensing to track range shifts due to climate change [3, 32]. Although we emphasize the need to mitigate snail-borne diseases, this study did not include parasite screening. Therefore, national-scale surveillance of these vectors should incorporate pathogen detection, prevalence studies, and genomic analyses to better understand the eco-evolutionary dynamics of snail-borne diseases. Future research should include parasite screening (e.g., S. mansoni, A. cantonensis) using molecular and microscopic techniques to refine public health risk assessments.

## Conclusions

This study demonstrates the utility of mitochondrial markers, particularly *COI* and 16S rRNA, in assessing the distribution and genetic structure of two invasive freshwater snails in Guangdong, China. *Physa acuta* shows widespread regional distribution and moderate mitochondrial diversity, suggesting geographic structuring rather than cryptic speciation. *B. straminea* exhibits limited genetic variation despite distinct morphological traits, such as red and black pigmentation. However, phylogenetic analysis revealed unresolved relationships within the Biomphalaria complex, underscoring the need for multi-locus genomic data to clarify species boundaries. These findings enhance our understanding of the genetic and morphological variation in these species and support future surveillance and taxonomic efforts in invasive snail populations.

## Supplementary Information

Supplementary material 1.

## Data Availability

The datasets used and/or analyzed during the current study are available in the Figshare repository at https://figshare.com/s/ea210260c4ee6e0d231d, with 10.6084/m9.figshare.29391926. Additional details are available from the corresponding author on reasonable request.

## Abbreviations

*COI*: Cytochrome *c* oxidase subunit I
ITS: Internal transcribed spacer
PCR: Polymerase chain reaction
eDNA: Environmental DNA
