# Supplementary material for: The morphology, genetic structure, and haplotype distribution of the invasive freshwater snails Biomphalaria straminea and Physa acuta in Guangdong, China
Source: Trop Med Health. 2025 Jul 28;53:98. doi: 10.1186/s41182-025-00780-y (PMC12302756; doi:10.1186/s41182-025-00780-y)
Supplement: Supplementary file 1 — Supplementary material 1. [file 41182_2025_780_MOESM1_ESM.pdf]

Supplementary information for

The morphology, genetic structure, and haplotype distribution of the invasive freshwater snails *Biomphalaria straminea* and *Physa acuta* in Guangdong, China

Ping He[1,2†], Yunyi Hu[2,3†], Jehangir Khan[4,5†], Yan Huang[2,3†], Zhanhong Yuan[2,3], Benjamin Sanogo[6], Du Gao[7], Jun Liu[8], De Wu[8], Jingdiao Chen[8], Zhongdao Wu[2,3], Song Liang[9*], Xi Sun[2,3*], and Datao Lin[2,3*]

[1]Medical Department of Xizang Minzu University, Xianyang, China. [2]Department of Parasitology, Key Laboratory of Tropical Disease Control (Ministry of Education), Zhongshan School of Medicine, Sun Yat-sen University, Guangzhou, China. [3]Chinese Atomic Energy Agency Center of Excellence on Nuclear Technology Applications for Insect Control, Provincial Engineering Technology Research Center for Diseases-vectors Control, Sun Yat-sen University, Guangzhou, China. [4]Department of Zoology, Abdul Wali Khan University Mardan, Pakistan. [5]Hainan General Hospital, Hainan Medical University, Haikou, China. [6]Laboratory of Parasitology, Institut National de Recherche en Sante Publique, Bamako, Mali. [7]Clinical laboratory, Dalian Dermatosis Hospital, Dalian, China. [8]Guangdong Provincial Center for Disease Control and Prevention, WHO Collaborating Centre for Surveillance, Research and Training of Emerging Infectious Diseases, Guangzhou, China. [9]Department of Environmental Health Sciences, School of Public Health & Health Sciences, University of Massachusetts, Amherst, USA.

[†]These authors contributed equally to this work.

*Correspondence: lindt5@mail.sysu.edu.cn (D.L.); sunxi2@mail.sysu.edu.cn (X.S.); songliang@umass.edu (S.L.)

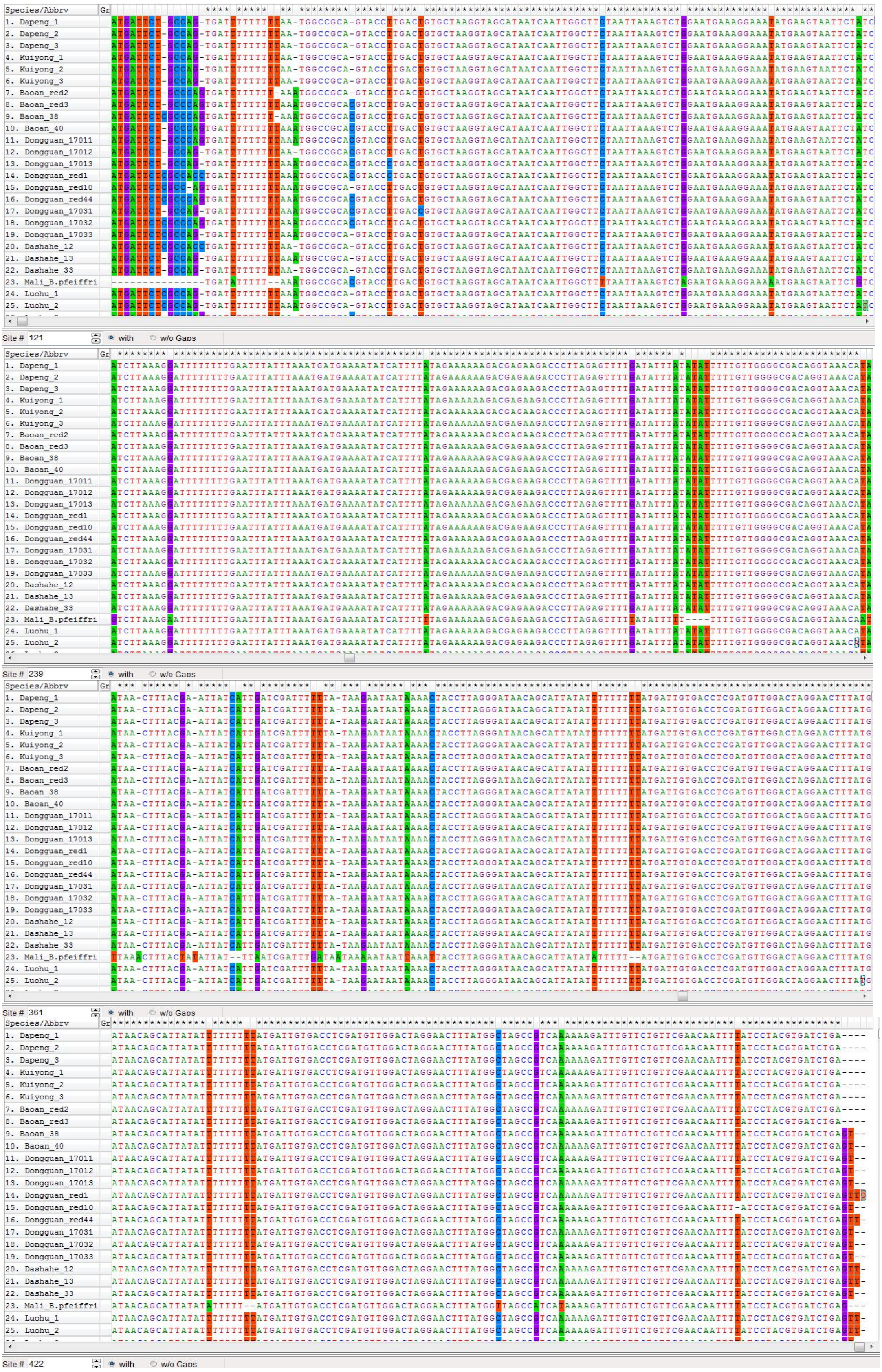

Fig. S1 Partial results of multiple alignments of 16S rRNA gene of *Biomphalaria* snails in Guangdong. * Represented the conserved region.

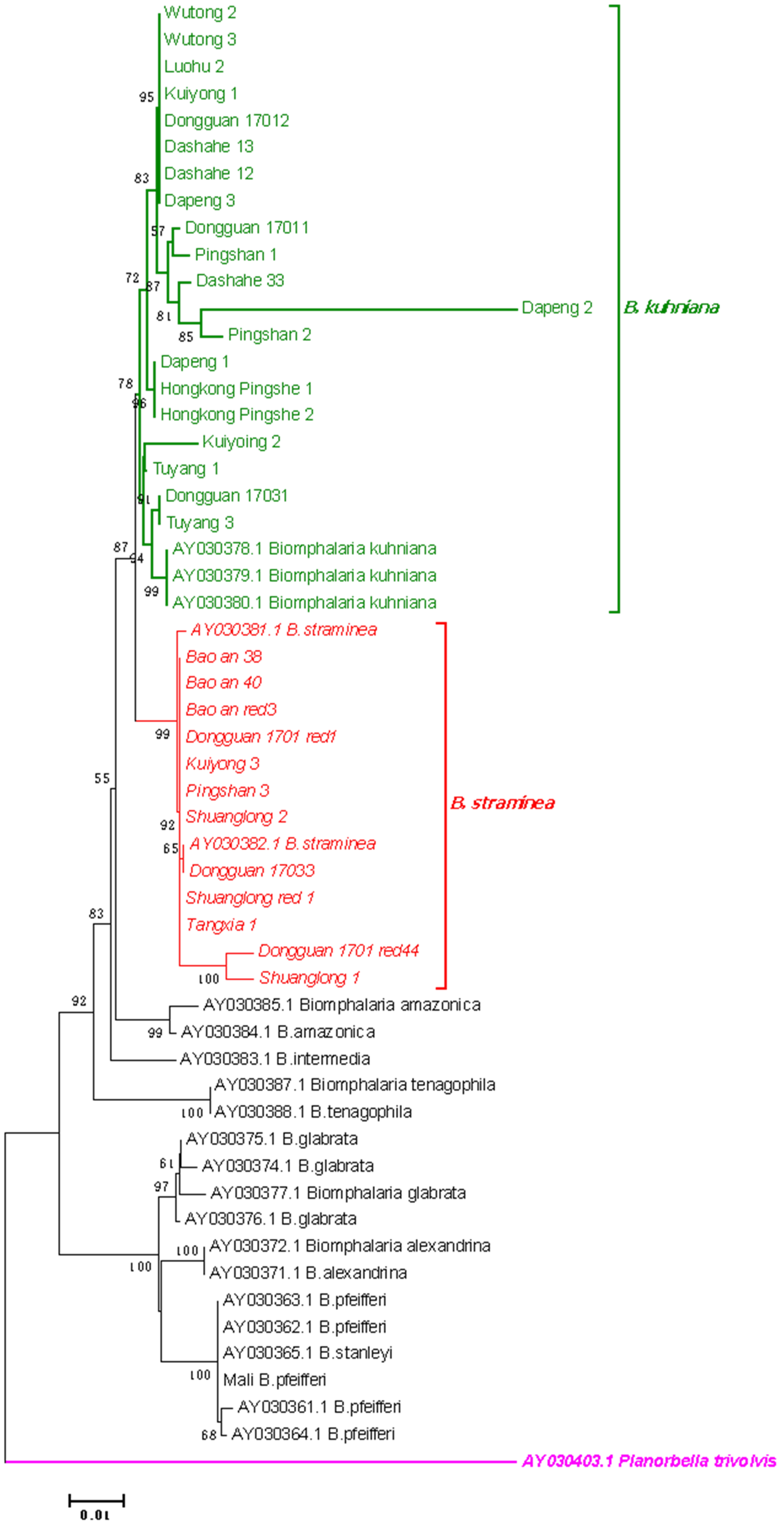

Fig. S2 Phylogenetic tree of ITS of *Biomphalaria* snails in Guangdong by Neighbor-Joining method

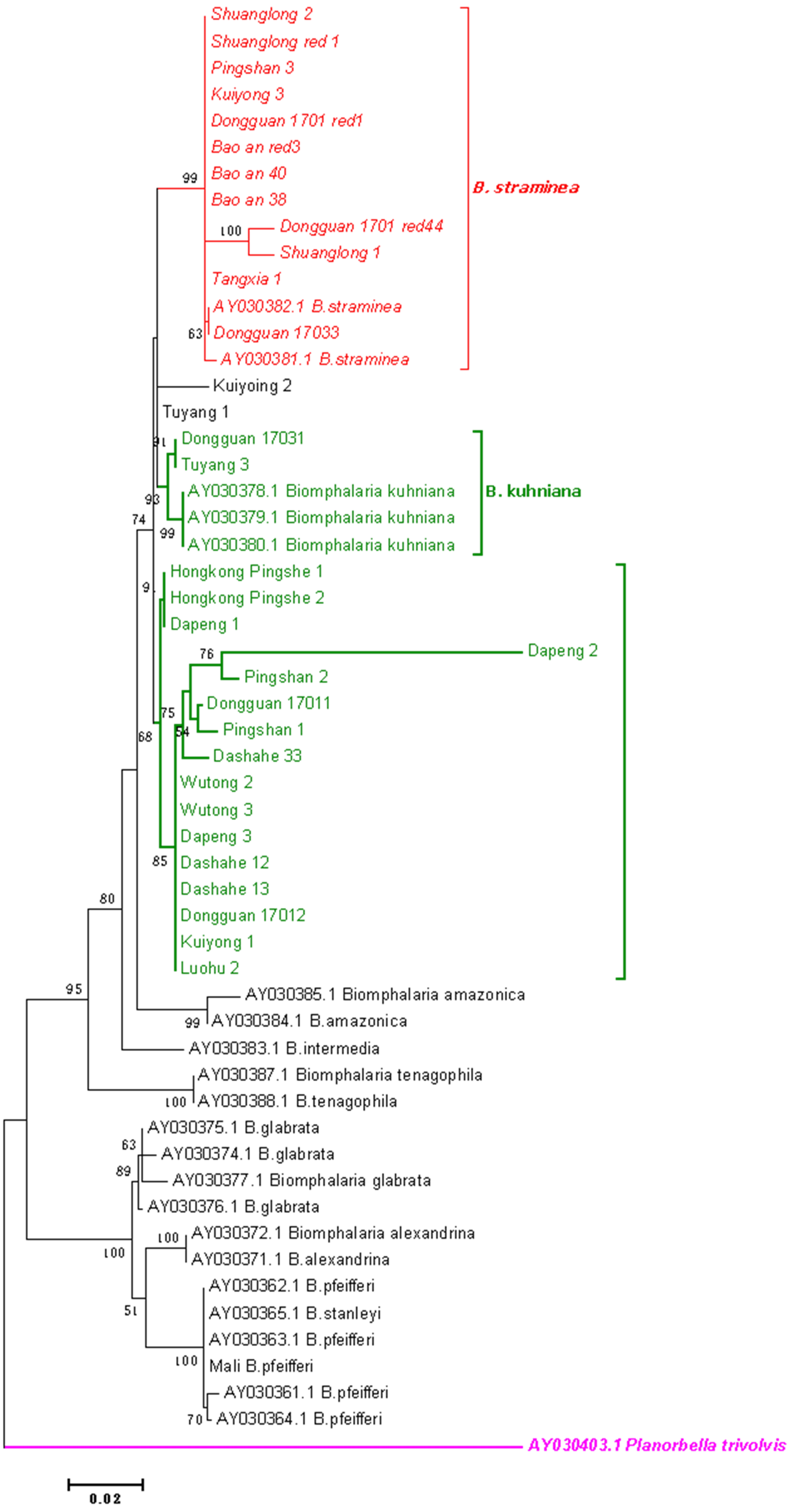

Fig. S3 Phylogenetic tree of ITS of *Biomphalaria* snails in Guangdong by Maximum likelihood method.

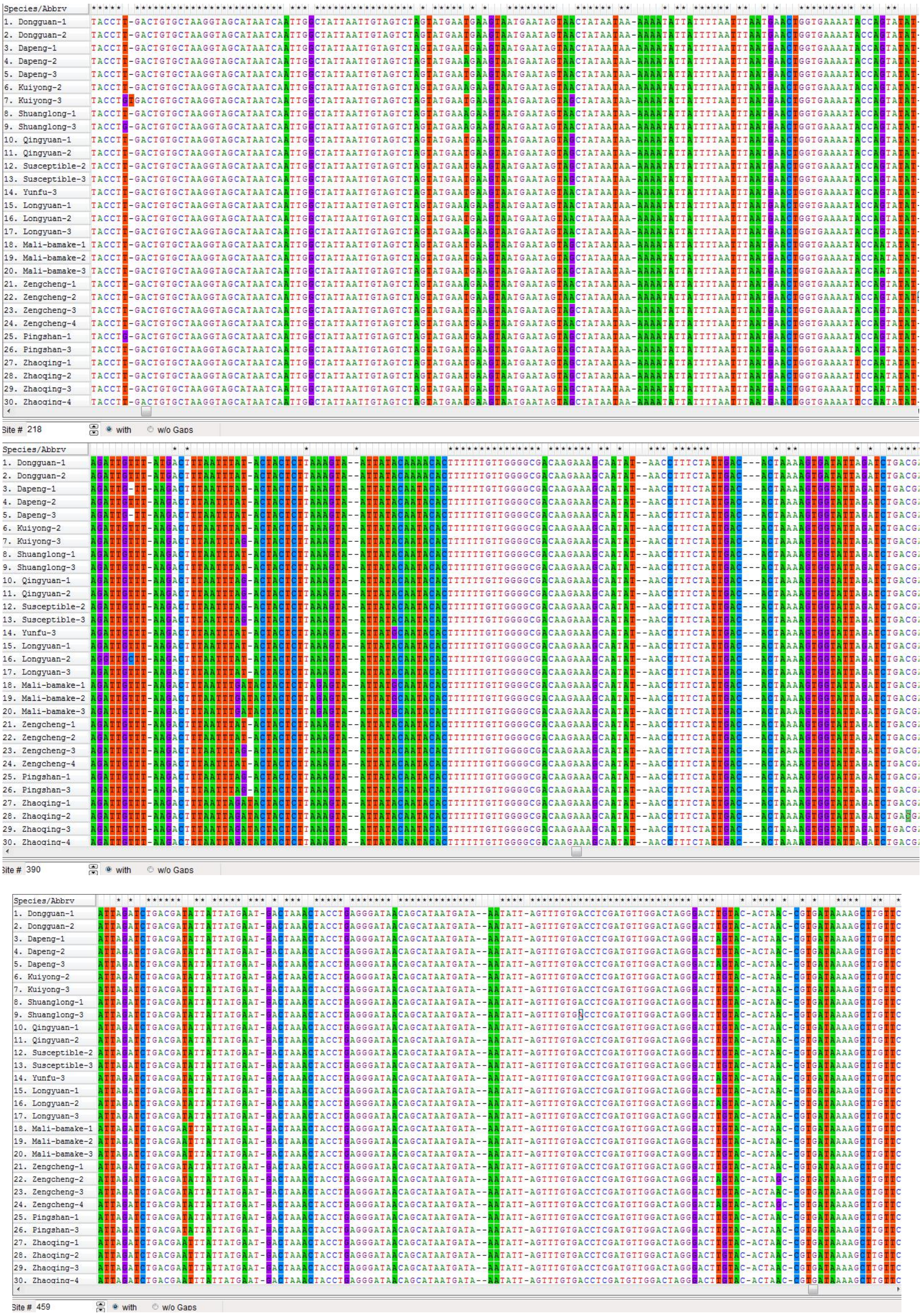

Fig. S4 Multiple alignments of 16S rRNA gene of *Physa acuta* in Guangdong. * Represented the conserved region.

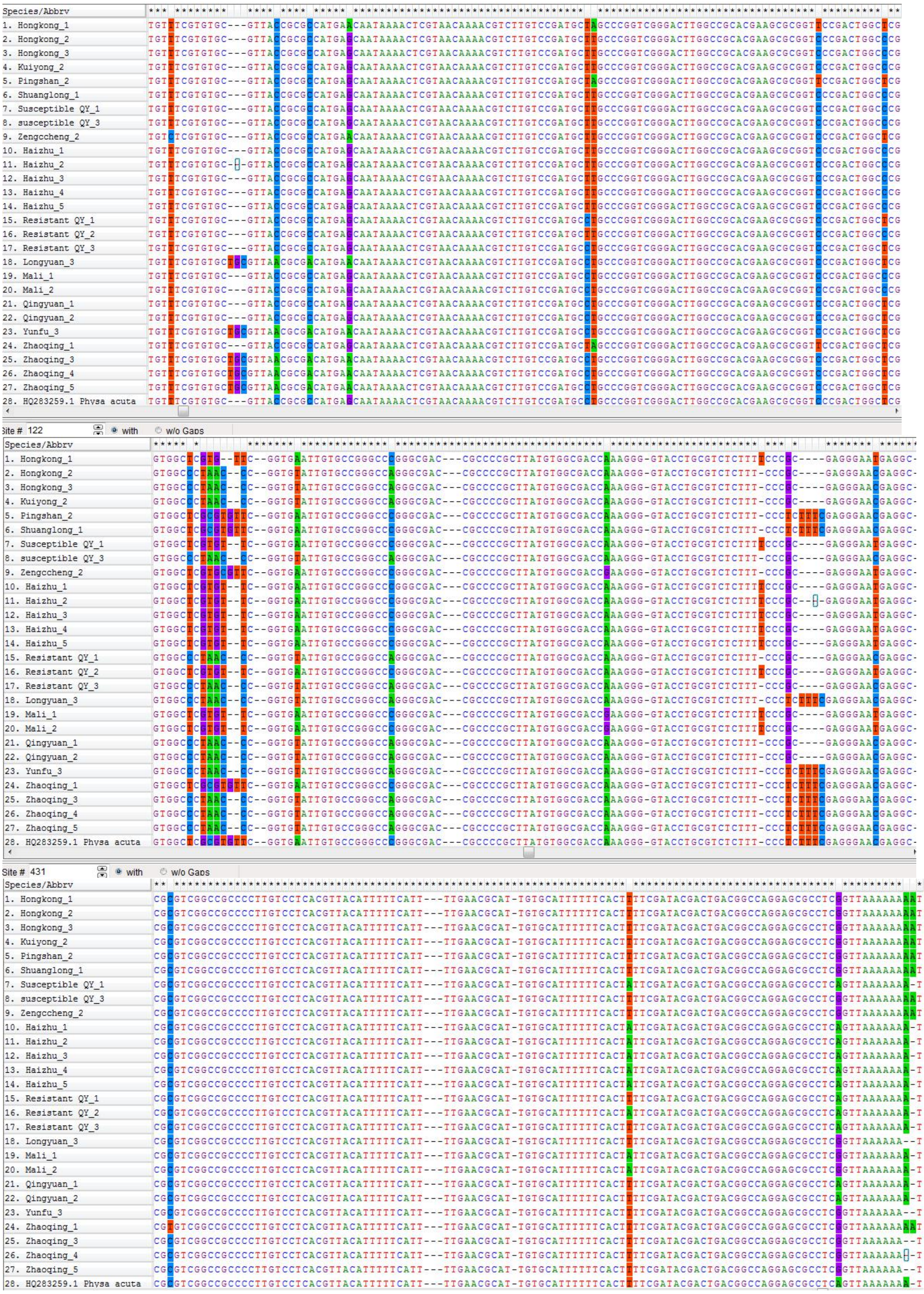

Fig. S5 Multiple alignments of ITS gene of *Physa acuta* in Guangdong. * Represented the conserved region.

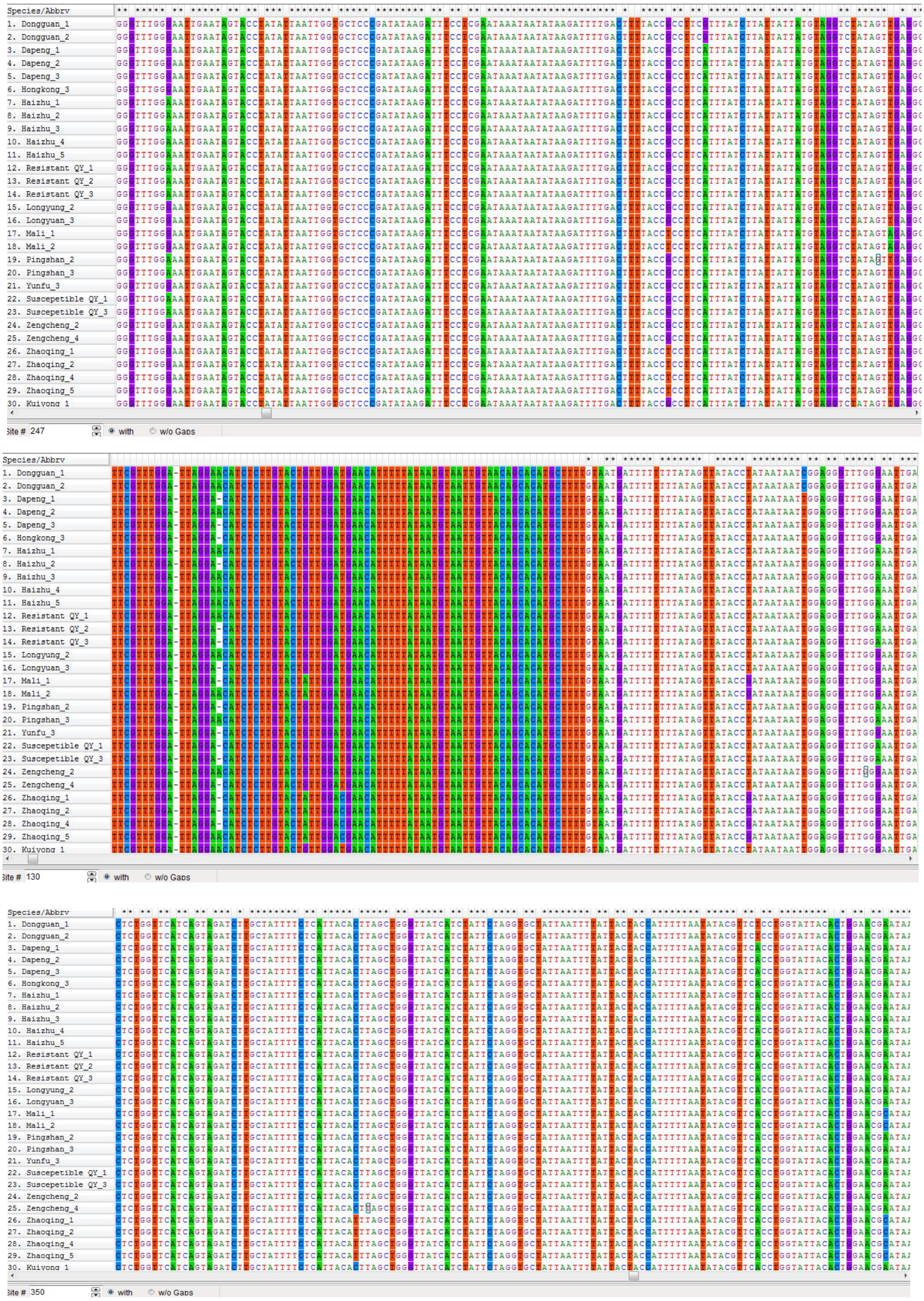

Fig. S6 Multiple alignments of *COI* gene of *Physa acuta* in Guangdong. * Represented the conserved region.

Table S1 PCR primers for *Biomphalaria* snail

Gene	Forward frimer	Reverse primer	Reference
COI	LCO1490:GGTCAACAAATCATAAAGATATTGG	HC02198:TAAACTTCAGGGTGACCAAAAAATCA	[9]
ITS	ETTS1:TGCTTAAGTTCAGCGGGT	ETTS2:TAACAAGGTTTCCGTAGGTGAA	[20]
16S rRNA	16Sar:CGCCTGTTTATCAAAAACAT	16Sbr:CCGGTCTGAACTCAGATCACGT	[9]

Table S2 PCR primers for *Physa acuta*

Gene	Forward frimer	Reverse primer	Reference
COI	TTGGGATCTGGTGTGGATTG	CCTCTTGGGTCATAGAACCT	FJ373016.1
ITS	GTGACGGCCTCGGATTGGTC	TTGGCTGCGGTTCTTCATCG	HQ283259.1
16S rRNA	CGCCTGTTTATCAAAAACAT	CCGGTCTGAACTCAGATCACGT	[9]
18S rRNA	TAAGTTCACACTGTCCCATGG	AGTCCCGTATTGTTATTTTTCG	L78905.1
28S rRNA	ACGTCTGCGTCGCCTTGTGG	GAGCGGCTACCTTTGGCACC	DQ256738.1

Table S3 PCR reaction condition for primers of *Biomphalaria* snails

Reaction process	Temperature	Time
pre-denaturation	95℃	5 min
denaturation	95℃	35 sec
annealing	53.9℃	35 sec
extension	72℃	30 sec
cycles	35	
extension	72℃	10min

Table S4 PCR reaction condition for 18S rRNA、28S rRNA and ITS primers of *Physa acuta*

Reaction process	Temperature	Time
pre-denaturation	95℃	5 min
denaturation	95℃	50 sec
annealing	55℃	35 sec
extension	72℃	50 sec
cycles	35	
extension	72℃	10 min

Table S5 PCR reaction condition for 16S rRNA and *COI* primers of *Physa acuta*

Reaction process	Temperature	Time
pre-denaturation	94℃	4 min
denaturation	94℃	30 sec
annealing	51℃	30 sec
extension	72℃	45 sec
cycles	35	
extension	72℃	10 min
